# Treatment Patterns and Outcomes in Patients with Advanced Biliary Tract Cancers Treated with Gemcitabine-Based Chemotherapy: A Retrospective Study

**DOI:** 10.3390/cancers17020305

**Published:** 2025-01-18

**Authors:** Farshid Dayyani, Heide A. Stirnadel-Farrant, Jenny Hu, Yian Lin, Nehemiah Kebede, Stephen J. Valerio, Daniel H. Ahn

**Affiliations:** 1 Division of Hematology/Oncology and Chao Family Comprehensive Cancer Center, University of California, Irvine, 200 S Manchester Avenue, Orange, CA 92868, USA; 2 Oncology Outcomes Research, AstraZeneca, Cambridge CB2 8PA, UK; heide.stirnadel-farrant@astrazeneca.com; 3 Oncology Data & Analytics, AstraZeneca, Gaithersburg, MD 20878, USA; jenny.hu@astrazeneca.com (J.H.); nehemiah.kebede@astrazeneca.com (N.K.); 4 Oncology Biometrics, Oncology Research & Development, AstraZeneca, South San Francisco, CA 94080, USA; ylin83@alumni.jh.edu; 5 United States Medical Affairs, AstraZeneca, Gaithersburg, MD 20878, USA; stephen.valerio@astrazeneca.com; 6 Division of Medical Oncology, Mayo Clinic, Phoenix, AZ 85054, USA; ahn.daniel@mayo.edu

**Keywords:** real-world outcomes, advanced biliary tract cancer, gemcitabine-based chemotherapy

## Abstract

**Background:** Historically, the standard of care for advanced biliary tract cancers (aBTCs) was gemcitabine plus cisplatin (GemCis). Immunotherapy plus GemCis is now recommended as a first-line treatment for aBTCs. Whether patients can tolerate eight cycles of GemCis in clinical practice, as per the Advanced Biliary Cancer (ABC)-02 study, remains to be assessed. We performed a retrospective observational cohort study to assess real-world treatment patterns and overall survival (OS) in patients with de novo or recurrent aBTCs treated with first-line gemcitabine-based chemotherapy in the United States. **Methods:** This retrospective observational cohort study used Optum’s de-identified Market Clarity Data (Market Clarity). Adults diagnosed with de novo or recurrent aBTCs in the United States who began first-line gemcitabine-based chemotherapy from January 2016–March 2022 were identified and followed from index until death, the end of continuous enrolment, or the end of study period. Treatment patterns and OS were assessed. **Results:** Overall, 559 patients were included (de novo, *n* = 462; recurrent, *n* = 97). GemCis was the most common first-line therapy received (de novo: 73.8%; recurrent: 57.7%). Most patients received approximately five cycles of GemCis; median (95% CI) time to discontinuation was 4.6 (4.3–5.1) months. Most patients died over the follow-up period (de novo: 70.3%; recurrent: 62.9%). Median OS (95% CI) was 14.2 (12.1–16.1) months (de novo) and 18.5 (15.6–26.9) months (recurrent). **Conclusions:** GemCis was the most common first-line therapy received during the study period; most patients were unable to receive eight cycles of GemCis. Survival was limited over the follow-up period, highlighting the need for new treatments for aBTCs. Future studies are warranted to understand the real-world impact of first-line immunotherapy plus GemCis for patients with aBTCs.

## Simple Summary

Historically, the standard of care for advanced biliary tract cancers (aBTCs) was chemotherapy (gemcitabine plus cisplatin [GemCis]). More recently, durvalumab and pembrolizumab (types of immunotherapies) have been used in combination with GemCis. Whether patients can tolerate eight cycles of GemCis in clinical practice, as per the Advanced Biliary Cancer (ABC)-02 study, remains to be assessed. This study evaluated chemotherapy treatment patterns inpatients with aBTCs in clinical practice in the United States, and the effectiveness of chemotherapy as a first-line treatment. Results showed that GemCis was the most common first-line treatment in patients with aBTCs. Most patients were unable to receive eight cycles of GemCis. Overall, 69% of patients died during our study, and median overall survival was approximately 15 months. The results highlight the limited efficacy of chemotherapy as a first-line treatment for aBTCs. Future studies are warranted to understand the impact of first-line treatment with immunotherapy plus GemCis for patients with aBTCs.

## 1. Introduction

Biliary tract cancers (BTCs) are a group of heterogeneous malignancies that include intrahepatic cholangiocarcinoma, extrahepatic cholangiocarcinoma, gallbladder cancer, and ampulla of Vater cancer [1]. In the United States, BTCs have been reported to occur at an age-standardised rate of 5.0% per 100,000 person-years [2]. The incidence of BTCs has continued to rise in the United States, primarily due to increased cases of intrahepatic cholangiocarcinoma [3].

Treatment for BTC depends on the stage of disease at diagnosis. For patients with resectable BTCs, the standard of care is curative intent therapy with surgery followed by adjuvant chemotherapy [4]. However, disease recurrence is common, with over 54.0% of patients with BTCs experiencing recurrence within 5 years following resection [5,6,7,8,9]. BTCs are typically aggressive, with non-specific symptoms in the early stages [1,10]. As a result, most patients are diagnosed at advanced or metastatic stages, where curative surgery is not feasible and the prognosis is poor [1].

Historically, the first-line standard of care for advanced BTCs (aBTCs) was gemcitabine (1000 mg/m^2^) plus cisplatin (25 mg/m^2^) (GemCis), based on findings from the Phase 3 Advanced Biliary Cancer (ABC)-02 study (NCT00262769) [11]. Several Phase 2 and 3 studies have evaluated targeted therapies for the first-line treatment of aBTCs, including cediranib, erlotinib, cetuximab, panitumumab, ramucirumab, and merestinib; however, none were able to improve survival when compared with gemcitabine-based chemotherapy [12,13,14,15,16].

Immune checkpoint inhibitors plus GemCis have now been established as the new standard of care, based on findings from the Phase 3 clinical trials TOPAZ-1 (NCT03875235) and KEYNOTE-966 (NCT04003636) [17,18,19,20]. Results from these studies were the first to demonstrate that adding immunotherapy (durvalumab and pembrolizumab, respectively) to GemCis could significantly improve overall survival (OS) compared with GemCis alone for patients with aBTCs [19,20].

GemCis is administered to patients with aBTCs based on findings from the ABC-02 study, in which GemCis was administered for eight cycles [11]. However, whether patients can tolerate eight cycles of GemCis within a real-world setting remains to be assessed.

As the treatment landscape for aBTCs evolves, there is also a need to understand treatment patterns and outcomes among real-world patients who are more likely to reflect broader practice populations compared with more selected clinical trial patient populations. Once available, real-world studies of first-line gemcitabine-based chemotherapy will provide a bench-mark for immunotherapy data and inform clinical decision-making and patient care.

This study aimed to assess real-world treatment patterns and OS in patients with de novo or recurrent aBTCs treated with first-line gemcitabine-based chemotherapy in the United States, in order toprovide critical insights into the practical challenges and limitations of standard therapies. 

## 2. Materials and Methods

### 2.1. Data Source

A retrospective observational cohort study was performed with patients diagnosed with de novo or recurrent advanced biliary tract cancers (aBTCs) from the United States. This study used Optum’s de-identified Market Clarity Data (Market Clarity), a database that deterministically links medical and pharmacy claims with electronic health record data from providers across the continuum of care in the United States. This study used de-identified data that complied with the requirements of the Health Insurance Portability and Accountability Act. Ethics Committee approval was not required due to this study’s retrospective nature and the use of de-identified data.

### 2.2. Study Population

The study population included adult patients (≥18 years of age) with de novo or recurrent aBTCs who initiated first-line gemcitabine-based chemotherapy between 1 January 2016 and 31 March 2022 (study period). The study period reflected an environment before the approval of durvalumab plus GemCis in September 2022 or pembrolizumab plus GemCis in October 2023 for treating locally advanced or metastatic BTCs (durvalumab plus GemCis) or locally advanced unresectable or metastatic BTCs (pembrolizumab plus GemCis) in the United States [17,18]. This ensured that only patients treated with gemcitabine-based chemotherapy were assessed before the approval of immunotherapy for aBTCs. The initiation of first-line gemcitabine-based chemotherapy following a diagnosis of BTC was designated as the index date. Treatment was expected to have been initiated soon after the index date, minimising the potential for immortal time bias.

Patients were required to have a diagnosis of BTC in the 12-month period before the index date. A diagnosis of BTC was defined as ≥2 diagnosis codes for BTC occurring 1–90 days apart in the electronic health record or claims (inpatient or non-diagnostic outpatient), using the International Classification of Diseases, Ninth or Tenth Revision, Clinical Modification (ICD-9-CM or ICD-10-CM) criteria. Continuous medical and pharmacy plan enrolment was required for ≥12 months before and for ≥3 months following the index date. First-line gemcitabine-based chemotherapy was required to have been administered for ≥6 weeks.

Patients were stratified into two cohorts based on whether they had de novo or recurrent BTC. Patients in the de novo cohort had not undergone surgical resection for BTC before the initiation of first-line gemcitabine-based chemotherapy. Patients in the recurrent cohort had undergone surgical resection (cholecystectomy, bile duct excision, hepatic resection, liver transplant, lymphadenectomy, or pancreatoduodenectomy) with or without adjuvant chemotherapy before the initiation of first-line gemcitabine-based chemotherapy.

### 2.3. Study Variables

Patient demographics and tumour characteristics were assessed on the index date. Comorbidities and risk factors, clinical signs and symptoms, and laboratory measures were assessed in the 12-month period before the index date. Comorbidities and risk factors for BTCs were identified using ICD-9-CM or ICD-10-CM codes and included fatty liver disease (nonalcoholic steatohepatitis/nonalcoholic fatty liver), liver cirrhosis, obesity, diabetes mellitus, inflammatory bowel disease, and viral hepatitis. Child–Pugh scores were poorly captured in our cohort. Albumin–bilirubin (ALBI) scores were used as an alternative measure of liver function because they could be calculated based on available laboratory values and were not limited by the lack of information on ascites and encephalopathy. ALBI scores were calculated using baseline albumin (measured in g/L) and bilirubin (measured in μmol/L) values that were assessed within eight weeks of one another, using the following formula: (log10 bilirubin × 0.66) + (albumin × −0.085). Patients were categorised based on their ALBI grade using the following ALBI scores: ALBI score ≤ −2.60 (ALBI grade 1), >−2.60 to ≤−1.39 (ALBI grade 2), and >−1.39 (ALBI grade 3), with a higher ALBI grade indicating greater liver function impairment [21].

Pre-index treatments were assessed between the earliest recorded diagnosis of BTC and the index date. Treatment patterns, including duration of treatment, first-, second-, or third-line treatment received, and treatment holidays, were assessed during the follow-up period (defined as the time from the index date until death, the end of continuous enrolment, or the end of the study period, whichever occurred first). The duration of treatment was estimated from the date of the first and last treatment administration. A line of therapy regimen was defined as all medications received within 30 days of initiating first-line gemcitabine-based chemotherapy for aBTCs. A line of therapy continued until the earliest of the following: end of the follow-up period, switch, or permanent treatment discontinuation. A switch was defined as initiating a new systemic therapy more than 30 days from starting a line of treatment. Discontinuation was defined as the permanent cessation of all therapy, indicated by a ≥60-day gap without any treatment before the end of the follow-up period. A treatment holiday was defined as a ≥30-day gap without any treatment administration between the start and the end dates of a line of therapy.

Real-world time to treatment discontinuation was calculated as the time from starting a line of therapy to discontinuation or death. An event was defined as permanent treatment discontinuation, treatment switch, or death while receiving treatment. Patients who did not have an event were censored at the end of continuous enrolment or the end of the data cut, whichever occurred first.

Real-world time to next treatment was calculated as the time from starting a line of therapy until the initiation of a subsequent line of therapy or death. An event was defined as treatment switch or death without switching to the next line of therapy. Patients who remained on or discontinued therapy and were alive at the end of the follow-up were censored at the end of continuous enrolment or data cut, whichever occurred first.

Real-world overall survival (OS) was calculated as the time from the start of first-line therapy to death from any cause. An event was defined as death from any cause. Our study aimed to estimate real-world OS among all patients with aBTC who received at least one line of therapy. We did not intend to estimate real-world OS only while patients were on the treatment. Therefore, patients with aBTC who discontinued first-line therapy were considered at risk of experiencing the event of interest (i.e., death). Patients were considered to be lost to follow-up if no additional encounters or claims were recorded in the Market Clarity database. In such cases, these patients were censored at their last known alive date (the latest of the service date from the administrative claims data and the last encounter date from the electronic health record).

Subgroup analyses were performed to assess real-world OS in all patients stratified by the number of first-line gemcitabine-based chemotherapy cycles received (≤8 vs. >8 cycles). Treatment cycles were identified based on unique gemcitabine administration dates observed during first-line treatment. The earliest administration of gemcitabine first-line treatment was considered the start of the first treatment cycle (cycle 1). A new cycle was defined based on the administration of gemcitabine ≥19 days from the start of a cycle.

Multivariate analyses were performed to explore how specific baseline demographics and clinical characteristics impacted the time to discontinuation of first-line gemcitabine-based chemotherapy and real-world OS. The following baseline demographic and clinical characteristics were included in the multivariate models: age at index; gender; race; BTC subtype; alcoholic liver disease; hepatitis B virus; hepatitis C virus; metabolic dysfunction-associated steatotic liver disease or metabolic dysfunction-associated steatohepatitis; biliary obstructive conditions (biliary obstruction, cholangitis, insertion/exchange of biliary stent, or jaundice); cardiovascular disease; diabetes mellitus; liver cirrhosis; obesity; ALBI grade; alanine aminotransferase level; aspartate aminotransferase level; and International Normalised Ratio. Baseline covariates were selected based on their clinical relevance and availability in the database. The Kolmogorov-type supremum test was used to evaluate for the proportional hazards assumption for the Cox proportional hazard models. A base case analysis was performed using a multivariate Cox proportional hazards model, where patients without recorded values for categorical variables were assigned a value of “Missing”. A complete case sensitivity analysis was also performed, restricting the analysis to patients with non-missing values for all covariates. The following reference ranges were used to identify elevated laboratory measures: alanine aminotransferase (>40 U/L); aspartate aminotransferase (>40 U/L); and International Normalised Ratio (>1.1).

### 2.4. Statistical Analyses

This is a descriptive, non-comparative study; analyses were summarised overall and by patients with de novo or recurrent aBTCs. Continuous variables were summarised by the number of patients, median, first and third quartiles (Q1, Q3), and range. Categorical variables were summarised by frequency counts and percentages for each category. All time-to-event analyses were estimated using Kaplan–Meier methods [22], with the median time-to-event and related 95% confidence interval (CI) presented. CIs were calculated using Greenwood standard error of the median or other landmark point estimates. Results were not presented for variables where the number of patients was less than five to protect patient identity. Cohort identification and derivation of line of therapy were conducted using MySQL. Additional analyses were performed using SAS Software, Version 9.4 or higher (SAS Institute, Cary, NC, USA).

## 3. Results

### 3.1. Study Population

In total, 559 patients with advanced biliary tract cancers (aBTCs) met the study selection criteria, including 462 patients in the de novo cohort and 97 patients in the recurrent cohort (Figure S1).

Baseline demographics across study cohorts are shown in Table 1. Most patients were diagnosed with biliary tract cancer (BTC) between 2018 and 2022 (69.6%) and were White (77.6%), non-Hispanic (81.6%), from the Midwest or Northeast regions of the United States (67.4%), and had commercial insurance (48.0%). The median (range) age at index across study cohorts was 65.0 (28.0–88.0) years. The recurrent cohort had a higher percentage of males (55.7%) than the de novo cohort (45.9%). The median time from the earliest BTC diagnosis date recorded in the electronic health record or claims to the index date was longer in the recurrent cohort (15.3 months) compared with the de novo cohort (0.9 months).

Clinical characteristics across study cohorts are presented in Table 2. Most patients (62.3%) were diagnosed with intrahepatic cholangiocarcinoma. Cardiovascular disease was the most common comorbidity/risk factor associated with BTC, recorded in 51.3% of patients. Overall, 16.8% of patients had metabolic dysfunction-associated steatotic liver disease or metabolic dysfunction-associated steatohepatitis (MASLD/MASH), and 38.8% of patients had biliary obstruction. For patients who had their albumin–bilirubin (ALBI) values recorded (*n* = 402), most (48.3%) had an ALBI grade of 2.

### 3.2. Pre-Index Treatment Patterns

Pre-index treatments administered between BTC diagnosis and the index date are shown in Table S1. In the recurrent cohort, most patients had undergone cholecystectomy or hepatic resection. Few patients in the recurrent cohort received adjuvant chemotherapy (without radiotherapy) or adjuvant radiotherapy (without chemotherapy) following any resection (cholecystectomy, bile duct excision, hepatic resection, lymphadenectomy, liver transplant, or pancreatoduodenectomy). Prior treatment with locoregional therapies, including embolisation (transarterial embolisation, transarterial chemoembolisation, or transarterial radioembolisation), ablation, and radiotherapy, was uncommon across study cohorts.

### 3.3. Post-Index Treatment Patterns

The median (Q1, Q3) duration of follow-up for assessment of treatment patterns was 10.5 (6.6, 17.8) months and 12.0 (6.6, 21.9) months in the de novo and recurrent cohorts, respectively. 

#### 3.3.1. First-Line Therapy

**Figure 1:**
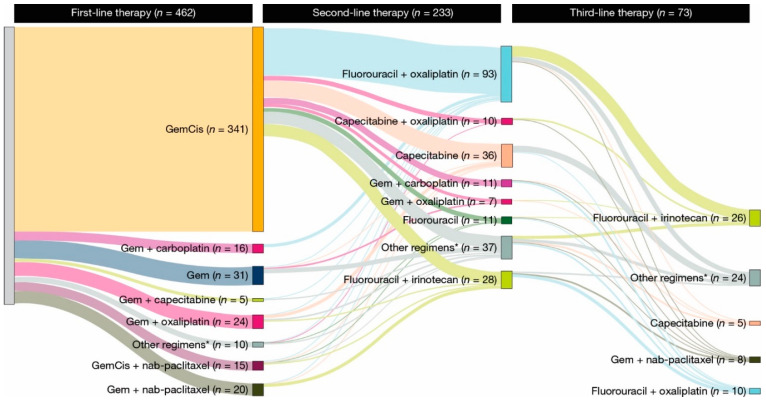
Treatment regimens administered during follow-up in patients with de novo advanced biliary tract cancer. * Regimens administered to <5 patients. Gem, gemcitabine; GemCis, gemcitabine plus cisplatin; nab, nanoparticle albumin-bound.

**Figure 2:**
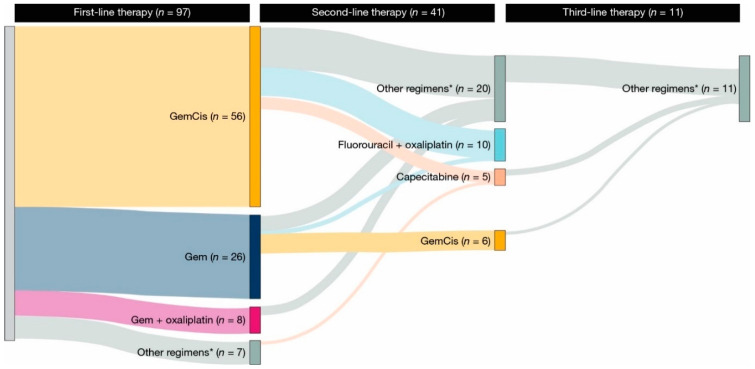
Treatment regimens administered during follow-up inpatients with recurrent advanced biliary tract cancer. * Regimens administered to <5 patients. Gem, gemcitabine; GemCis, gemcitabine plus cisplatin.

**Figure 3:**
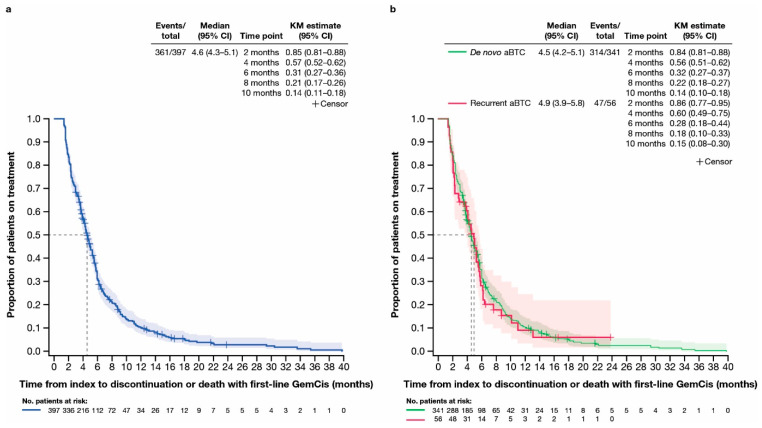
Time to discontinuation or death with first-line gemcitabine plus cisplatin. (**a**) Kaplan–Meier curve of time to discontinuation or death with first-line gemcitabine plus cisplatin in all patients included in this study. (**b**) Kaplan–Meier curves of time to discontinuation or death with first-line gemcitabine plus cisplatin in subgroups of patients with de novo or recurrent advanced biliary tract cancer. aBTC, advanced biliary tract cancer; CI, confidence interval; GemCis, gemcitabine plus cisplatin; KM, Kaplan–Meier.

Gemcitabine plus cisplatin (GemCis) was the most common type of first-line gemcitabine-based chemotherapy received in the de novo (Figure 1) and recurrent (Figure 2) aBTC cohorts. The median (95% CI) time to discontinuation or death with first-line GemCis was 4.6 (4.3–5.1) months for all patients (Figure 3a), 4.5 (4.2–5.1) months in the de novo cohort (Figure 3b), and 4.9 (3.9–5.8) months in the recurrent cohort (Figure 3b). In total, 32.0% of patients in the de novo cohort and 28.0% of patients in the recurrent cohort remained on first-line GemCis at 6 months, and 14.0% and 15.0% remained on first-line GemCis at 10 months, respectively (Figure 3b).

In total, 22.1% of patients in the de novo cohort and 27.8% of patients in the recurrent cohort had ≥1 treatment holiday during first-line gemcitabine-based chemotherapy (Table S2). The duration of treatment holidays by regimen type is shown in Table S3. Of the patients who received first-line gemcitabine-based chemotherapy, 49.0% switched to a new line of therapy, 34.5% permanently discontinued treatment, and 9.3% remained on treatment during the follow-up period (Table S2). Overall, 7.2% of patients died while receiving first-line gemcitabine-based chemotherapy (Table S2).

The median (95% CI) time to discontinuation or death with first-line gemcitabine-based chemotherapy was 4.4 (4.0–4.8) months in the de novo cohort and 4.6 (4.1–5.3) months in the recurrent cohort (Figure S2a). In total, 31.0% of patients in the de novo cohort and 30.0% of patients in the recurrent cohort remained on first-line gemcitabine-based chemotherapy at 6 months, and 14.0% and 18.0% remained on first-line gemcitabine-based chemotherapy at 10 months, respectively (Figure S2a).

Multivariate analyses were performed, using a Cox proportional hazards model, to explore how specific baseline demographics and clinical characteristics impacted the time to discontinuation of first-line gemcitabine-based chemotherapy. The base case analysis included all 559 patients, while the sensitivity analysis was restricted to 314 patients with non-missing values for all covariates (complete case). Of these, 507 patients in the base case analysis and 289 patients in the complete case analysis experienced an event, i.e., discontinued first-line gemcitabine-based chemotherapy. In the base case analysis, patients of “Other” race were more likely to discontinue first-line gemcitabine-based chemotherapy compared with patients who were White (Table S4). In the sensitivity analysis, patients with aspartate aminotransferase (AST) levels exceeding the reference range were more likely to discontinue first-line gemcitabine-based chemotherapy compared with patients who had normal AST levels (Table S5).

#### 3.3.2. Second-Line Therapy

Second-line therapy was received in 50.4% of patients in the de novo cohort and 42.3% of patients in the recurrent cohort (Table S2). The median (95% CI) time from initiation of first-line gemcitabine-based chemotherapy to second-line therapy or death was 7.9 (7.4–8.6) months in the de novo cohort and 11.5 (7.3–13.7) months in the recurrent cohort (Figure S3a). Fluorouracil plus oxaliplatin was the most common second-line regimen received across cohorts (Figure 1 and Figure 2). In total, 14.6% of patients in the de novo cohort had ≥1 treatment holiday during second-line therapy (Table S2). Data were unavailable for the number of patients in the recurrent cohort who had ≥1 treatment holiday during second-line therapy due to small patient numbers (Table S2). Of the patients who received second-line therapy, 39.8% permanently discontinued treatment and 30.7% switched to a new line of therapy during the follow-up period (Table S2). In total, 17.2% of patients died while receiving second-line therapy (Table S2). The median (95% CI) time to discontinuation or death with second-line therapy was 2.1 (1.8–2.6) months in the de novo cohort and 2.1 (1.5–4.4) months in the recurrent cohort (Figure S2b).

#### 3.3.3. Third-Line Therapy

Few patients in the de novo and recurrent cohorts received third-line therapy (Figure 1 and Figure 2). The most common third-line therapy received in the de novo cohort was fluorouracil plus irinotecan (Figure 1). Data were unavailable for the most common third-line therapy in the recurrent cohort due to small patient numbers (Figure 2). The median (95% CI) time from second-line to third-line therapy was 4.9 (4.1–6.0) months in the de novo cohort and 6.9 (4.9–13.8) months in the recurrent cohort (Figure S3b). Due to small patient numbers, data were unavailable for patients who had treatment holidays during third-line therapy, and the reasons for the end of third-line therapy were unavailable (Table S2). The median (95% CI) time to discontinuation or death with third-line therapy was 2.1 (1.4–3.0) months in the de novo cohort and 3.2 (1.6–not estimable) months in the recurrent cohort (Figure S2c).

### 3.4. Overall Survival

#### 3.4.1. Overall Survival Across Study Cohorts

**Figure 4:**
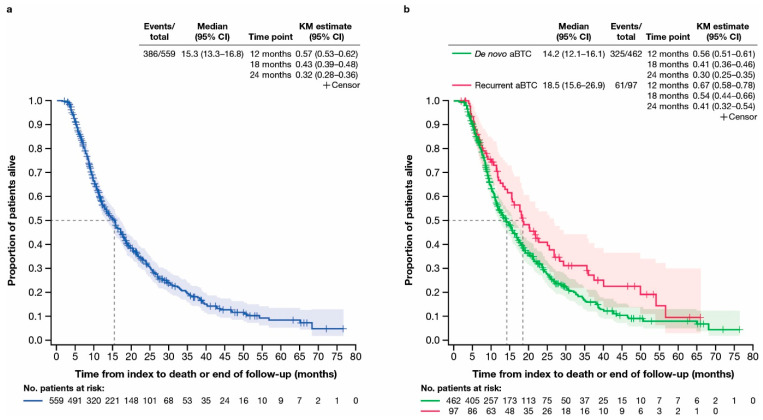
Time from index to death or end of follow-up. (**a**) Kaplan–Meier curve of time from index to death or end of follow-up for all patients included in this study. (**b**) Kaplan–Meier curves of time from index to death or end of follow-up in subgroups of patients with de novo or recurrent advanced biliary tract cancer. aBTC, advanced biliary tract cancer; CI, confidence interval; KM, Kaplan–Meier.

The median (Q1, Q3) duration of follow-up for overall survival (OS) was 11.5 (7.3, 21.2) months for all patients, 11.3 (7.3, 19.5) months in the de novo cohort, and 14.4 (7.3, 25.6) months in the recurrent cohort. Median OS (95% CI) was 15.3 (13.3–16.8) months for all patients (Figure 4a) and was longer in the recurrent (18.5 [15.6–26.9] months) versus the de novo cohort (14.2 [12.1–16.1] months) (Figure 4b). In the de novo and recurrent cohorts, 56.0% and 67.0% of patients were alive at 12 months, 41.0% and 54.0% were alive at 18 months, and 30.0% and 41.0% were alive at 24 months, respectively (Figure 4b). In total, 70.3% and 62.9% of patients died over the follow-up period in the de novo and recurrent cohorts, respectively.

Multivariate analyses were performed, using a Cox proportional hazards model, to explore how specific baseline demographics and clinical characteristics impacted real-world OS. The base case analysis included all 559 patients, while the sensitivity analysis was restricted to 314 patients with non-missing values for all covariates (complete case). Of these, 386 patients in the base case analysis and 232 patients in the complete case analysis experienced an event, i.e., died post-index. The base case analysis showed that patients with gallbladder cancer had a higher risk of death compared with patients with intrahepatic cholangiocarcinoma (Table S6). Furthermore, patients with an ALBI grade of 2 versus 1 and those exhibiting AST levels above the reference range also showed an elevated risk of death (Table S6). In the sensitivity analysis, an increased risk of death was found for patients with hepatitis C virus (HCV) compared with those without HCV and for patients with an ALBI grade of 2 or 3 versus 1 (Table S7).

#### 3.4.2. Overall Survival in All Patients by the Number of First-Line Gemcitabine-Based Chemotherapy Cycles Received

**Figure 5:**
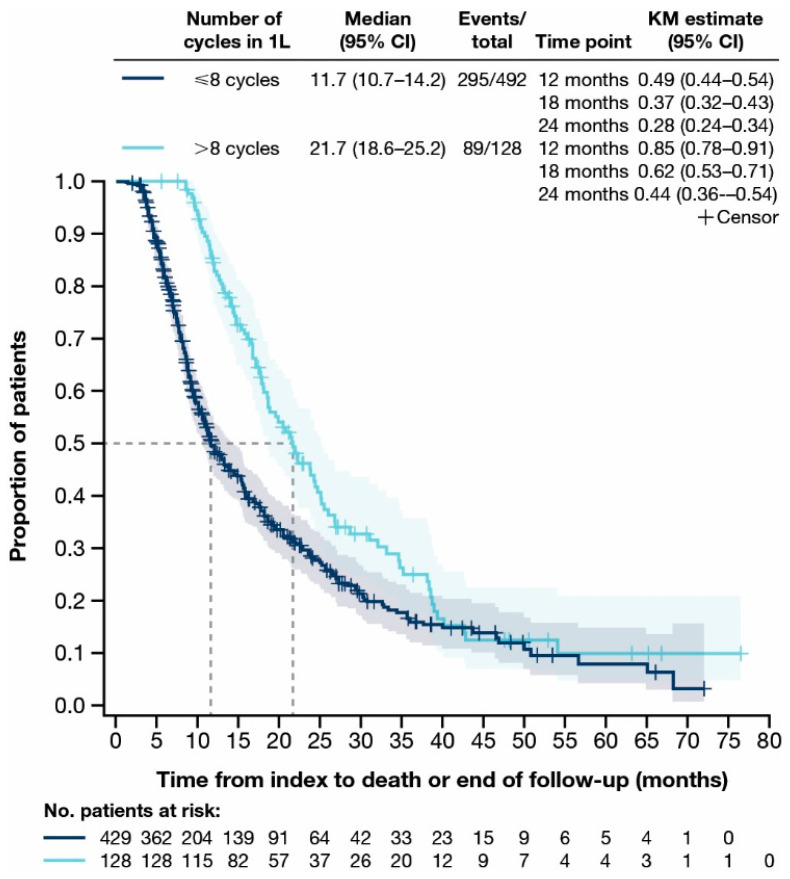
Time from index to death or end of follow-up for all patients with advanced biliary tract cancer stratified by the number of gemcitabine-based chemotherapy cycles received in first-line. Two patients treated with first-line gemcitabine monotherapy were excluded from this analysis as their gemcitabine records were within 7 days of each other. 1L, first-line; CI, confidence interval; KM, Kaplan–Meier.

The median (Q1, Q3) duration of follow-up for OS was 18.1 (13.0, 26.8) months for patients who received >8 cycles of first-line gemcitabine-based chemotherapy and 9.6 (6.2, 18.2) months for patients who received ≤8 cycles of first-line gemcitabine-based chemotherapy. Median OS (95% CI) was 21.7 (18.6–25.2) months for patients who received >8 cycles of first-line gemcitabine-based chemotherapy and 11.7 (10.7–14.2) months for patients who received ≤8 cycles of first-line gemcitabine-based chemotherapy (Figure 5). For patients who received >8 cycles of first-line gemcitabine-based chemotherapy, 85.0%, 62.0%, and 44.0% were alive at 12, 18, and 24 months, respectively (Figure 5). Most patients died over the follow-up period regardless of the number of cycles of first-line gemcitabine-based chemotherapy (≤8 cycles: *n* = 295/429 [68.8%]; >8 cycles: *n* = 89/128 [69.5%]).

## 4. Discussion

This retrospective study evaluated real-world treatment patterns and overall survival (OS) for patients with de novo or recurrent advanced biliary tract cancers (aBTCs) treated with first-line gemcitabine-based chemotherapy in the United States. Patient demographics and clinical characteristics were generally similar across cohorts and representative of other studies reporting real-world treatment patterns or outcomes for patients with aBTCs, providing confidence in our results [23,24].

Similar to other real-world studies on biliary tract cancers (BTCs), the most common first-line treatment administered to patients with de novo or recurrent aBTCs was gemcitabine plus cisplatin (GemCis) [23,24]. Other common first-line gemcitabine-based chemotherapies received in our study included gemcitabine monotherapy and gemcitabine plus oxaliplatin.

Almost half of the patients included in our study went on to receive second-line treatment. The most common second-line regimens received were fluorouracil plus oxaliplatin and capecitabine. Patients were treated with second-line therapy for approximately 2 months across cohorts, and most patients discontinued second-line therapy over the follow-up period. Few patients in our study received third-line therapy. While data were limited in the recurrent cohort due to small patient numbers, the most common third-line regimens in the de novo cohort included fluorouracil plus irinotecan, fluorouracil plus oxaliplatin, gemcitabine plus nanoparticle albumin-bound-paclitaxel, and capecitabine. Of note, the time to treatment discontinuation decreased across cohorts from first-line gemcitabine-based chemotherapy to third-line therapy. Similar findings have been reported in another real-world study that assessed treatment patterns in BTCs, indicating the limited effectiveness of treatment in later lines [24].

In clinical practice, GemCis is administered to patients with aBTCs based on findings from the ABC-02 study, in which GemCis was administered for eight cycles (i.e., approximately 6 months) [11]. In our analysis, first-line GemCis was discontinued at a median of 4.6 months (i.e., after approximately five cycles). The most common reason for discontinuing first-line gemcitabine-based chemotherapy was due to switching to a new line of therapy, most likely due to progressive disease or a lack of tolerability. In an additional subgroup analysis, median OS (95% CI) was longer for patients who received >8 cycles (21.7 [18.6–25.2] months) versus ≤8 cycles (11.7 [10.7–14.2] months) of first-line gemcitabine-based chemotherapy. However, most patients (>68.0%) died over the follow-up period, regardless of the number of cycles of first-line gemcitabine-based chemotherapy received. These results are valuable, as they indicate that most patients with de novo or recurrent aBTCs cannot receive eight cycles of GemCis in clinical practice. Our study did not capture why patients could not receive eight cycles of GemCis. However, it is expected that this may be due to disease progression, tolerability issues, or death. Additional studies may be warranted to explore these findings in more detail.

Few studies have previously assessed real-world treatment patterns and outcomes for patients with aBTCs [24,25,26]. A systematic literature review of treatment patterns and outcomes for patients with unresectable, advanced, or metastatic BTCs from Australia, Canada, Germany, France, South Korea, and the United Kingdom showed similar results to our study and reported the most common second-line regimens as a combination of systemic therapy, gemcitabine-based chemotherapy, and fluoropyrimidine-based chemotherapy [25]. However, it must be noted that the systematic literature review was performed in different geographical locations from our study and did not include a detailed patient flow diagram, making comparisons across studies difficult [25]. Compared with our study, another study of treatment patterns for patients with BTCs using Merative MarketScan administrative claims databases reported a lower incidence of second- and third-line therapy use (20.9% vs. 49.0% and 7.1% vs. 15.0%); however, there was limited overlap between the most common types of second- and third-line therapies received across studies [24]. Several reasons may explain these differences, including using different patient databases, calendar years, and study periods for patient selection [24]. A further retrospective study in patients with advanced cholangiocarcinoma in the United States who experienced failure of first-line gemcitabine or 5-fluorouracil therapy reported gemcitabine, fluorouracil, and capecitabine as the most common second- and third-line treatments received [26]. The results from our study cannot be directly compared with this study for several reasons. Different study periods were used for the analysis in the other study (2007–2019), with our research reflecting a more current treatment landscape based on a cut-off date of 2022. Patients with gallbladder cancer were included in our study, whereas the other study focussed exclusively on patients with cholangiocarcinoma. The other study only included patients with failure of gemcitabine-based or 5-fluorouracil-based therapy [26]. In contrast, our study included all patients treated with first-line gemcitabine-based treatment, regardless of whether they failed first-line therapy. In addition, the other study excluded patients alive at the end of the follow-up period, with no evidence of initiating a next line of therapy [26]. This may have led to immortal time bias and limited the comparison of results with our study. Lastly, the other study used Optum’s de-identified Clinformatics^®^ Data Mart Database (Clinformatics^®^), which differs from Market Clarity used in our study [26]. Data Mart is limited to administrative claims, does not include linkage to electronic health records, and is subject to known limitations inherent within claims data. In contrast, Market Clarity comprises electronic health record data linked to administrative claims, provides a more comprehensive capture of diagnoses and laboratory assessments, and incorporates claims data from Optum-affiliated payors and third-party sources. Irrespective of the differences noted across studies, collectively, the results suggest a lack of standard of care for patients with aBTCs who progress beyond first-line treatment and the need for more effective treatment options within this setting.

Assessment of real-world OS showed that median OS (95% CI) was 15.3 (13.3–16.8) months for all patients with aBTCs, with most patients in both cohorts dying over the follow-up period. In the de novo and recurrent cohorts, 56.0% and 67.0% of patients were alive at 12 months, respectively.

The base case analysis showed that patients with gallbladder cancer had a higher risk of death compared with patients with intrahepatic cholangiocarcinoma. Additionally, patients with an albumin-bilirubin (ALBI) grade of two versus one and those exhibiting aspartate aminotransferase levels above the reference range also showed an elevated risk of death. In the sensitivity analysis, an increased risk of death was noted for patients with hepatitis C virus and those with an ALBI grade of two or three. However, these results should be interpreted with caution due to the small patient numbers in some subgroups, necessitating further validation in larger patient cohorts.

The real-world OS rates reported in our study were generally longer, and OS rates at 12 months were similar to or higher than those previously reported in other real-world studies in aBTCs [23,24,27]. In a real-world study of patients with locally advanced or metastatic BTCs in the United States, median OS (95% CI) and 12-month OS rates were 8.1 (7.4–8.9) months and 33.9% with first-line GemCis, 5.2 (4.3–6.5) months and 19.2% with first-line gemcitabine monotherapy, and 8.4 (6.6–10.4) months and 35.1% for all other first-line regimens [27]. In another real-world study of patients with aBTCs in the United States, median OS was 12.7 months (95% CI not reported), and 32.5% of patients were alive at the end of the follow-up period (mean of 11.9 months) [24]. Furthermore, in a real-world study that assessed survival outcomes in Canadian patients with advanced or metastatic BTCs, median OS (95% CI) was 11.0 (10.4–11.7) months, and the 12-month OS rate was 47.0% [23]. Several reasons may contribute to the variation in OS reported between studies, including differences in study cohort eligibility criteria, treatment regimens received, study periods, follow-up durations, and potential differences in the distinct levels of care patients received due to different healthcare systems [23,24,27]. For example, though the analysis by Danese et al. was conducted on a US population, the study cohort included patients through 2015 [27]. This current analysis included more recent patients when additional second-line agents such as pemigatinib, infigratinib, and ivosidenib had been approved, which could have led to improved OS. These factors also need to be taken into consideration when considering how the data presented here would extrapolate to other geographies, particularly as treatment patterns, including subsequent therapies, would be expected to differ. Despite this, taken together, these results highlight the limited OS benefits of first-line gemcitabine-based chemotherapy regimens for patients with aBTCs.

The treatment landscape for aBTCs has recently evolved with the inclusion of immunotherapy. TOPAZ-1 was the first Phase 3 study to show that combining durvalumab with GemCis could improve OS compared with GemCis alone in participants with aBTCs [19]. At the pre-planned interim analysis of TOPAZ-1, durvalumab plus GemCis demonstrated a significant improvement in OS versus placebo plus GemCis, with an OS hazard ratio of 0.80 (95.0% CI 0.66–0.97; *p* = 0.021, significance threshold 0.03) [19]. Additionally, durvalumab plus GemCis showed a significant improvement in progression-free survival and an improvement in objective response rate [19]. Based on the results of TOPAZ-1, durvalumab plus GemCis was the first immunotherapy-based combination to be approved in the United States to treat adults with locally advanced, metastatic, or unresectable BTC [28]. Since the approval of durvalumab plus GemCis, recent findings from the KEYNOTE-966 trial also support combining immunotherapy with GemCis for participants with aBTCs [20]. While the study designs of TOPAZ-1 and KEYNOTE-966 were generally similar, they allowed different durations of gemcitabine therapy [19,20]. In TOPAZ-1, participants received GemCis in combination with placebo or durvalumab for up to eight cycles, and gemcitabine maintenance was not allowed [19]. This differs from KEYNOTE-966, where there was no limit to the number of gemcitabine cycles that could be received [20]. Taken together, the results from our study suggest that most patients in the real world cannot tolerate eight cycles of GemCis and are, therefore, unlikely to go on to receive an extended duration of gemcitabine therapy. While long-term follow-up of immunotherapy in aBTCs in the real world is awaited, recent findings published in 2024 from a real-world retrospective multicentre study support the benefit of durvalumab plus GemCis in aBTCs in clinical practice, reporting a median OS with durvalumab plus GemCis and GemCis of 14.8 and 11.2 months, respectively [29]. Moving forward, it will be important to understand the long-term impact of immunotherapy on real-world outcomes for people living with aBTCs.

This study was limited by its retrospective and observational nature; findings depended on the accuracy and completeness of medical charts and administrative claims. We acknowledge that the requirement of continuous enrolment in a medical and pharmacy plan may have introduced selection bias, potentially excluding patients with lower socioeconomic status and limited access to care. This, in turn, could have impacted findings on treatment patterns and outcomes. For example, outcomes could be expected to be affected by patients who had access to insurance over a continuous period compared with uninsured patients or those not enrolled in continuous care. Therefore, our study findings may not be generalisable to uninsured patients and those covered by Medicare fee-for-service (Part A & B). Continuous enrolment was critical for meeting the aims of our study. This approach helped us accurately identify the study population of interest (de novo aBTC and recurrent BTC) and ensured a more comprehensive capture of patients’ baseline characteristics and treatment patterns during the follow-up period. Requiring continuous enrolment was also essential to mitigate potential misclassification bias. Specifically, it helped prevent the misclassification of patients with recurrent aBTC, as patients with de novo aBTC and ensured the accurate identification of treatments administered during the follow-up period. There was also a risk of lost-to-follow-up bias, but this was mitigated through patient censoring at the last known visit. In this study, initiating first-line gemcitabine-based chemotherapy was used as a proxy for aBTCs. While the definition of aBTCs was guided by clinical knowledge, there may be uncertainty as to whether patients truly had aBTCs. Additionally, this study only included patients who received gemcitabine-based chemotherapies, and patients who received the best supportive care following a diagnosis of aBTCs were not included. The electronic health record component of Market Clarity contains information derived from physician notes. However, there was limited capture of the rationale for initiating and discontinuing medications in this study, and the reasons for treatment holidays were unavailable. Analyses that may be of clinical interest, such as how treatment holidays impacted OS or other outcomes, could not be performed and would be an important area for future research. Patients were only followed until loss of continuous enrolment, death, or the end of this study period (whichever occurred first). Thus, this study did not capture any treatments administered following the loss of enrolment. Data were presented from patients across the United States and the impact of healthcare disparities (e.g., regions and socioeconomic groups) was not assessed. The Market Clarity database poorly captured clinically meaningful variables that may be prognostic of treatment discontinuation and survival, such as Eastern Cooperative Oncology Group performance status, cancer stage at index, Child–Pugh score, disease progression, and tumour response. This lead to a high level of missing data for specific variables of interest and limited the multivariate analyses.

## 5. Conclusions

In conclusion, the findings from this retrospective study showed that the most common first-line treatment administered to patients with de novo or recurrent advanced biliary tract cancers (aBTCs) was gemcitabine plus cisplatin (GemCis). The median time to discontinuation of first-line GemCis was less than 5 months across cohorts, and most patients had discontinued first-line GemCis after 6 months. Assessment of real-world overall survival showed that most patients with de novo (70.3%) or recurrent (62.9%) aBTCs died over the follow-up period, highlighting the need for new treatment options in this setting. As the treatment landscape evolves, further studies are warranted to understand the impact of immunotherapy on real-world outcomes for people with aBTCs.

## Figures and Tables

**Table 1:** Baseline demographics.

Baseline Demographics	De Novo Advanced BTC (*n* = 462)	Recurrent Advanced BTC (*n* = 97)	Total (*n* = 559)
Year of advanced BTC diagnosis (index year), *n* (%)			
2016	69 (14.9)	16 (16.5)	85 (15.2)
2017	68 (14.7)	17 (17.5)	85 (15.2)
2018	90 (19.5)	15 (15.5)	105 (18.8)
2019	96 (20.8)	16 (16.5)	112 (20.0)
2020	67 (14.5)	15 (15.5)	82 (14.7)
2021–2022	72 (15.6)	18 (18.6)	90 (16.1)
Age at index, years, median (range)	64.0 (28.0–88.0)	68.0 (39.0–87.0)	65.0 (28.0–88.0)
Age categories at index, *n* (%)			
18–74 years	365 (79.0)	73 (75.3)	438 (78.4)
≥75 years	97 (21.0)	24 (24.7)	121 (21.6)
Gender, *n* (%)			
Female	250 (54.1)	43 (44.3)	293 (52.4)
Male	212 (45.9)	54 (55.7)	266 (47.6)
Race, *n* (%)			
White	356 (77.1)	78 (80.4)	434 (77.6)
African American	54 (11.7)	6 (6.2)	60 (10.7)
Other/unknown	52 (11.3)	13 (13.4)	65 (11.6)
Ethnicity, *n* (%)			
Not Hispanic	383 (82.9)	73 (75.3)	456 (81.6)
Hispanic	23 (5.0)	6 (6.2)	29 (5.2)
Other/unknown	56 (12.1)	18 (18.6)	74 (13.2)
Region, *n* (%)			
Midwest	183 (39.6)	36 (37.1)	219 (39.2)
Northeast	128 (27.7)	30 (30.9)	158 (28.3)
South	103 (22.3)	16 (16.5)	119 (21.3)
West or other/unknown	48 (10.4)	15 (15.5)	63 (11.3)
Insurance type, *n* (%)			
Commercial	227 (49.1)	41 (42.3)	268 (48.0)
Medicare	160 (34.6)	41 (42.3)	201 (36.0)
Medicaid	39 (8.4)	5 (5.2)	44 (7.9)
Other/unknown	36 (7.8)	10 (10.3)	46 (8.2)
Time from earliest BTC diagnosis date recorded in electronic health record or claims to the index date, months, median (Q1, Q3)	0.9 (0.5, 1.6)	15.3 (6.2, 28.2)	1.2 (0.6, 3.3)

BTC, biliary tract cancer; Q, quartile.

**Table 2:** Clinical characteristics.

Clinical Characteristics	De Novo Advanced BTC (*n* = 462)	Recurrent Advanced BTC (*n* = 97)	Total (*n* = 559)
Site of primary tumour, *n* (%)			
Intrahepatic cholangiocarcinoma	306 (66.2)	42 (43.3)	348 (62.3)
Gallbladder cancer	55 (11.9)	24 (24.7)	79 (14.1)
Extrahepatic cholangiocarcinoma	45 (9.7)	14 (14.4)	59 (10.6)
Other *	56 (12.1)	17 (17.5)	73 (13.1)
Comorbid conditions of interest and risk factors of BTC ^†^, *n* (%)			
Biliary obstruction	176 (38.1)	41 (42.3)	217 (38.8)
Insertion or exchange of biliary stent	138 (29.9)	31 (32.0)	169 (30.2)
Jaundice	141 (30.5)	27 (27.8)	168 (30.1)
Cardiovascular disease	225 (48.7)	62 (63.9)	287 (51.3)
Diabetes	158 (34.2)	38 (39.2)	196 (35.1)
Liver cirrhosis	51 (11.0)	7 (7.2)	58 (10.4)
Obesity	186 (40.3)	35 (36.1)	221 (39.5)
Conditions associated with the aetiology of BTC ^†^, *n* (%)			
Viral hepatitis	27 (5.8)	NA	NA
MASLD/MASH	75 (16.2)	19 (19.6)	94 (16.8)
Other ^‡^	19 (4.1)	NA	NA
Albumin value recorded, *n* (%)	334 (72.3)	69 (71.1)	403 (72.1)
Albumin, g/dL, median (range)	3.7 (1.4–5.0)	3.8 (2.0–4.7)	3.7 (1.4–5.0)
Bilirubin value recorded, *n* (%)	336 (72.7)	68 (70.1)	404 (72.3)
Bilirubin, mg/dL, median (range)	0.7 (0.2–16.5)	0.6 (0.2–13.7)	0.7 (0.2–16.5)
ALBI grade, *n*/*N* (%)			
Grade 1	127/334 (38.0)	29/68 (42.6)	156/402 (38.8)
Grade 2	162/334 (48.5)	32/68 (47.1)	194/402 (48.3)
Grade 3	45/334 (13.5)	7/68 (10.3)	52/402 (12.9)
International Normalised Ratio recorded, *n* (%)	277 (60.0)	52 (53.6)	329 (58.9)
International Normalised Ratio, median (range)	1.1 (0.9–3.9)	1.1 (0.9–1.9)	1.1 (0.9–3.9)
Alanine aminotransferase value recorded, *n* (%)	337 (72.9)	69 (71.1)	406 (72.6)
Alanine aminotransferase value, median (range)	33.0 (6.0–624.0)	30.0 (8.0–351.0)	32.5 (6.0–624.0)
Aspartate aminotransferase value recorded, *n* (%)	335 (72.5)	69 (71.1)	404 (72.3)
Aspartate aminotransferase value, median (range)	43.0 (11.0–502.0)	31.0 (11.0–292.0)	40.0 (11.0–502.0)
CA 19-9 recorded, *n* (%)	246 (53.2)	43 (44.3)	289 (51.7)
CA 19-9, U/mL, median (range)	158.0 (0.1–200,478.0)	72.7 (2.0–20,000.0)	121.0 (0.1–200,478.0)

* Includes patients with ampulla of Vater cancer, malignant neoplasms of overlapping sites in the biliary tract, and unspecified malignant neoplasms of the biliary tract. ^†^ Not mutually exclusive. ^‡^ Includes alcoholic liver disease, drug-induced liver injury, and autoimmune hepatitis. ALBI, albumin-bilirubin; BTC, biliary tract cancer; CA 19-9, cancer antigen 19-9; MASH, metabolic dysfunction-associated steatohepatitis; MASLD, metabolic dysfunction-associated steatotic liver disease; *n*, number of patients; *N*, number of evaluable patients; NA, not available.

## Data Availability

Data sharing is not available. Data were obtained from Optum Market Clarity and are not able to be shared by the authors.

## Supplementary Materials

The following supporting information can be downloaded at https://www.mdpi.com/article/10.3390/cancers17020305/s1: Table S1. Pre-index treatments administered between biliary tract cancer diagnosis and the index date; Table S2. Treatment regimens administered during follow-up, treatment holidays, and reasons for end of follow-up; Table S3. Treatment holidays observed by regimen type during first-line gemcitabine-based chemotherapy; Table S4. Base case analysis: Cox proportional hazards model for discontinuation of first-line therapy in patients with aBTCs treated with gemcitabine-based chemotherapies; Table S5. Sensitivity analysis: Cox proportional hazards model for discontinuation of first-line therapy in patients with aBTCs treated with gemcitabine-based chemotherapies; Table S6. Base case analysis: Cox proportional hazards model for real-world OS in patients with aBTCs treated with gemcitabine-based chemotherapies; Table S7. Sensitivity analysis: Cox proportional hazards model for real-world OS in patients with aBTCs treated with gemcitabine-based chemotherapies; Figure S1. Patient selection criteria; Figure S2. Time from index to discontinuation or death by line of therapy in subgroups of patients with de novo or recurrent advanced biliary tract cancer; Figure S3. Time from initiation of first-line gemcitabine-based chemotherapy or second-line therapy to subsequent line of therapy or death in subgroups of patients with de novo or recurrent advanced biliary tract cancer.
