# Supplementary material for: Treatment Patterns and Outcomes in Patients with Advanced Biliary Tract Cancers Treated with Gemcitabine-Based Chemotherapy: A Retrospective Study
Source: Cancers (Basel). 2025 Jan 18;17(2):305. doi: 10.3390/cancers17020305 (PMC11764298; doi:10.3390/cancers17020305)
Supplement: Supplementary file 1 [file cancers-17-00305-s001.zip › cancers-3388832-supplementary.pdf]

SUPPLEMENTARY MATERIALS

Table S1. Pre-index treatments administered between biliary tract cancer diagnosis and the index date.

	De novo **advanced BTC (*n* = 462)**	**Recurrent advanced BTC (*n* = 97)**	**Total (*n* = 559)**
**Surgical resection type, *n* (%)**			
Cholecystectomy	0	58 (59.8)	58 (10.4)
Hepatic resection	0	44 (45.4)	44 (7.9)
Pancreatoduodenectomy	0	24 (24.7)	24 (4.3)
Lymphadenectomy	0	23 (23.7)	23 (4.1)
Bile duct excision	0	15 (15.5)	15 (2.7)
Liver transplant	0	NA	NA
**Use of any adjuvant chemotherapy following resection, *n* (%)**			
Adjuvant chemotherapy	0	15 (15.5)	15 (2.7)
Adjuvant radiotherapy	0	5 (5.2)	5 (0.9)
Adjuvant chemoradiotherapy	0	NA	NA

**Type of adjuvant chemotherapies administered +/- radiotherapy following resection*, *n* (%)**	*n* = 0	*n* = 17	*n* = 17
Gemcitabine	0	12 (70.6)	12 (70.6)
Capecitabine	0	5 (29.4)	5 (29.4)
Cisplatin	0	NA	NA
Fluorouracil	0	NA	NA
Oxaliplatin	0	NA	NA
**Locoregional therapies administered between the earliest BTCs diagnosis and index*, *n* (%)**			
Any embolisation[†]	13 (2.8)	7 (7.2)	20 (3.6)
Any ablation	5 (1.1)	6 (6.2)	11 (2.0)
Radiotherapy (no embolisation within ± 30 days and no resection within ± 12 weeks)	19 (4.1)	12 (12.4)	31 (5.6)

NA, data are not presented in order to preserve patient identity due to low patient numbers.

*Not mutually exclusive; [†]Transarterial embolisation, transarterial chemoembolisation, or transarterial radioembolisation.

BTC, biliary tract cancer; NA, not available.

Table S2. Treatment regimens administered during follow-up, treatment holidays, and reasons for end of follow-up.

	***De novo* advanced BTC (*n* = 462)**	**Recurrent advanced BTC (*n* = 97)**	**Total (*n* = 559)**
First-line regimen received, *n* (%)	462 (100.0)	97 (100.0)	559 (100.0)
Number of treatment holidays during first-line therapy, *n* (%)			
0	360 (77.9)	70 (72.2)	430 (76.9)
1	66 (14.3)	22 (22.7)	88 (15.7)
2+	36 (7.8)	5 (5.2)	41 (7.3)
Reason for end of first-line therapy, *n* (%)			
Permanently discontinued therapy	159 (34.4)	34 (35.1)	193 (34.5)
Switched to a new line of therapy	233 (50.4)	41 (42.3)	274 (49.0)
Died while on line of therapy regimen	33 (7.1)	7 (7.2)	40 (7.2)
Remained on treatment	37 (8.0)	15 (5.5)	52 (9.3)
Second-line regimen received, *n* (%)	233 (50.4)	41 (42.3)	274 (49.0)

Number of treatment holidays during second-line therapy, *n* (%)			
0	199 (85.4)	36 (87.8)	235 (85.8)
1	26 (11.2)	NA	NA
2+	8 (3.4)	NA	NA
Reason for end of second-line therapy, *n* (%)			
Permanently discontinued therapy	92 (39.5)	17 (41.5)	109 (39.8)
Switched to a new line of therapy	73 (31.3)	11 (26.8)	84 (30.7)
Died while on line of therapy regimen	43 (18.5)	9 (22.0)	47 (17.2)
Remained on treatment	25 (10.7)	NA	NA
Third-line regimen received, *n* (%)	73 (15.8)	11 (11.3)	84 (15.0)
Number of treatment holidays during third-line therapy, *n* (%)			
0	NA	NA	NA
1	NA	NA	NA
2+	NA	NA	NA
Reason for end of third-line therapy, *n* (%)			
Permanently discontinued therapy	29 (39.7)	NA	NA
Switched to a new line of therapy	19 (26.0)	NA	NA
Died while on line of therapy regimen	11 (15.1)	NA	NA
Remained on treatment	14 (19.2)	NA	NA

NA, data are not presented in order to preserve patient identity due to low patient numbers.

BTC, biliary tract cancer; NA, not available.

Table S3. Treatment holidays observed by regimen type during first-line gemcitabine-based chemotherapy.

Regimen	Patients receiving the regimen, *n* (%)	Patients with ≥1 treatment holiday, *n* (%)	Median (Q1, Q3) duration of treatment holidays per number of treatment holidays, days
***De novo* advanced BTC**			
GemCis	341	73 (21.4)	46.0 (36.0, 66.0)
Gemcitabine monotherapy	31	6 (19.4)	49.3 (35.0, 56.0)
Gemcitabine plus nab-paclitaxel	20	9 (45.0)	46.0 (36.0, 70.0)
GemCis plus nab-paclitaxel	15	5 (33.3)	47.5 (38.0, 98.0)
***Recurrent* advanced BTC**			
GemCis	56	15 (26.8)	42.0 (35.0, 111.0)
Gemcitabine monotherapy	26	10 (38.5)	42.8 (35.0, 59.0)

Treatment holidays observed by regimen type during first-line treatment are shown for *n* ≥5, only.

BTC, biliary tract cancer; GemCis, gemcitabine plus cisplatin; nab, nanoparticle albumin-bound; Q, quartile.

Table S4. Base case analysis: Cox proportional hazards model for discontinuation of first-line therapy in patients with aBTCs treated with gemcitabine-based chemotherapies

		**Multivariate model**		
**Parameter**	**Categories**	**Hazard ratio (95% CI)**	***p*-value**	**Type 3 *p*-value**
Age at index (years)	Continuous	1.01 (1.00–1.02)	0.2049	0.2049
Gender (reference: Male)	Female	0.93 (0.77–1.12)	0.4318	0.4318
Race (reference: White)	African American	1.03 (0.76–1.38)	0.8616	0.1224
	Other	1.36 (1.01–1.83)	0.0406	
Site of primary tumour (reference: Intrahepatic cholangiocarcinoma)	Extrahepatic cholangiocarcinoma	0.86 (0.63–1.18)	0.3581	0.5032
	Gallbladder cancer	1.09 (0.84–1.43)	0.5105	
	Ampulla of Vater cancer / BTC not otherwise specified	1.15 (0.83–1.59)	0.4061	
	Multiple sites	0.76 (0.43–1.33)	0.3347	
**Comorbid conditions of interest and risk factors of BTC**				
Biliary obstructive conditions (reference: No)	Yes	0.96 (0.78–1.18)	0.6857	0.6857
Cardiovascular disease (reference: No)	Yes	1.12 (0.92–1.35)	0.2561	0.2561
Diabetes (reference: No)	Yes	0.90 (0.73–1.09)	0.2785	0.2785
Liver cirrhosis (reference: No)	Yes	0.97 (0.67–1.39)	0.8532	0.8532
Obesity (reference: No)	Yes	0.92 (0.76–1.13)	0.4351	0.4351
**Conditions associated with the aetiology of BTC**				
Alcoholic liver disease (reference: No)	Yes	0.62 (0.35–1.12)	0.1122	0.1122

Hepatitis B virus (reference: No)	Yes	1.34 (0.55–3.23)	0.5190	0.5190
Hepatitis C virus (reference: No)	Yes	1.07 (0.64–1.79)	0.8036	0.8036
MASLD/MASH (reference: No)	Yes	0.93 (0.72–1.20)	0.5567	0.5567
**Laboratory measures**				
ALBI grade (reference: Grade 1)	Grade 2	1.00 (0.78–1.28)	0.9845	0.9878
	Grade 3	0.99 (0.68–1.46)	0.9741	
	Missing	0.77 (0.18–3.25)	0.7171	
Alanine aminotransferase (reference: Normal)	Higher than reference range	0.82 (0.62–1.08)	0.1524	0.1836
	Missing	0.42 (0.10–1.75)	0.2315	
Aspartate aminotransferase (reference: Normal)	Higher than reference range	1.29 (0.99–1.68)	0.0607	0.0840
	Missing	3.38 (0.45–25.4)	0.2363	
International Normalised Ratio (reference: Normal)	Higher than reference range	1.09 (0.83–1.43)	0.5511	0.7937
	Missing	0.98 (0.77–1.26)	0.8885	

aBTC, advanced biliary tract cancer; ALBI, albumin-bilirubin; BTC, biliary tract cancer; CI, confidence interval; MASH, metabolic dysfunction-associated steatohepatitis; MASLD, metabolic dysfunction-associated steatotic liver disease.

Table S5. Sensitivity analysis: Cox proportional hazards model for discontinuation of first-line therapy in patients with aBTCs treated with gemcitabine-based chemotherapies

		Multivariate model		
**Parameter**	**Categories**	**Hazard ratio (95% CI)**	***p*-value**	**Type 3 *p*-value**
Age at index (years)	Continuous	1.01 (1.00–1.02)	0.1063	0.1063
Gender (reference: Male)	Female	0.86 (0.66–1.11)	0.2407	0.2407
Race (reference: White)	African American	0.83 (0.57–1.23)	0.3591	0.3259
	Other	1.33 (0.81–2.17)	0.2602	
Site of primary tumour (reference: Intrahepatic cholangiocarcinoma)	Extrahepatic cholangiocarcinoma	0.78 (0.52–1.17)	0.2310	0.3330
	Gallbladder cancer	1.09 (0.74–1.61)	0.6650	
	Ampulla of Vater cancer / BTC not otherwise specified	0.88 (0.58–1.34)	0.5580	
	Multiple sites	0.50 (0.23–1.11)	0.0876	
**Comorbid conditions of interest and risk factors of BTC**				
Biliary obstructive conditions (reference: No)	Yes	1.13 (0.85–1.51)	0.3931	0.3931
Cardiovascular disease (reference: No)	Yes	1.10 (0.85–1.42)	0.4878	0.4878
Diabetes (reference: No)	Yes	0.95 (0.73–1.25)	0.7341	0.7341
Liver cirrhosis (reference: No)	Yes	0.99 (0.63–1.55)	0.9690	0.9690
Obesity (reference: No)	Yes	1.13 (0.86–1.47)	0.3830	0.3830
**Conditions associated with the aetiology of BTC**				
Alcoholic liver disease (reference: No)	Yes	0.88 (0.42–1.82)	0.7234	0.7234

Hepatitis B virus (reference: No)	Yes	1.09 (0.36–3.27)	0.8807	0.8807
Hepatitis C virus (reference: No)	Yes	0.91 (0.46–1.83)	0.8005	0.8005
MASLD/MASH (reference: No)	Yes	0.86 (0.62–1.19)	0.3634	0.3634
**Laboratory measures**				
ALBI grade (reference: Grade 1)	Grade 2	1.14 (0.85–1.52)	0.3942	0.6916
	Grade 3	1.08 (0.70–1.67)	0.7188	
Alanine aminotransferase (reference: Normal)	Higher than reference range	0.91 (0.67–1.24)	0.5586	0.5586
Aspartate aminotransferase (reference: Normal)	Higher than reference range	1.36 (1.01–1.82)	0.0418	0.0418
International Normalised Ratio (reference: Normal)	Higher than reference range	0.95 (0.71–1.26)	0.7071	0.7071

aBTC, advanced biliary tract cancer; ALBI, albumin-bilirubin; BTC, biliary tract cancer; CI, confidence interval; MASH, metabolic dysfunction-associated steatohepatitis; MASLD, metabolic dysfunction-associated steatotic liver disease.

Table S6. Base case analysis: Cox proportional hazards model for real-world OS in patients with aBTCs treated with gemcitabine-based chemotherapies

		Multivariate model		
**Parameter**	**Categories**	**Hazard ratio (95% CI)**	***p*-value**	**Type 3 *p*-value**
Age at index (years)	Continuous	1.01 (1.00–1.02)	0.0124	0.0124
Gender (reference: Male)	Female	0.93 (0.75–1.16)	0.5305	0.5305
Race (reference: White)	African American	0.73 (0.51–1.04)	0.0826	0.1386
	Other	1.14 (0.81–1.60)	0.4648	
Site of primary tumour (reference: Intrahepatic cholangiocarcinoma)	Extrahepatic cholangiocarcinoma	1.00 (0.70–1.41)	0.9811	0.0533
	Gallbladder cancer	1.61 (1.18–2.21)	0.0030	
	Ampulla of Vater cancer/ BTC not otherwise specified	1.02 (0.68–1.53)	0.9160	
	Multiple sites	1.17 (0.64–2.12)	0.6099	
**Comorbid conditions of interest and risk factors of BTC**				
Biliary obstructive conditions (reference: No)	Yes	0.93 (0.72–1.18)	0.5342	0.5342
Cardiovascular disease (reference: No)	Yes	0.86 (0.68–1.08)	0.1839	0.1839
Diabetes (reference: No)	Yes	0.97 (0.77–1.22)	0.7699	0.7699
Liver cirrhosis (reference: No)	Yes	1.15 (0.78–1.69)	0.4865	0.4865
Obesity (reference: No)	Yes	0.89 (0.71–1.12)	0.3225	0.3225
**Conditions associated with the aetiology of BTC**				
Alcoholic liver disease (reference: No)	Yes	0.82 (0.45–1.52)	0.5373	0.5373

Hepatitis B virus (reference: No)	Yes	2.07 (0.81–5.28)	0.1290	0.1290
Hepatitis C virus (reference: No)	Yes	1.57 (0.92–2.69)	0.1004	0.1004
MASLD/MASH (reference: No)	Yes	0.78 (0.58–1.06)	0.1077	0.1077
**Laboratory measures**				
ALBI grade (reference: Grade 1)	Grade 2	1.45 (1.09–1.94)	0.0115	0.0847
	Grade 3	1.49 (0.95–2.32)	0.0816	
	Missing	1.23 (0.29–5.20)	0.7785	
Alanine aminotransferase (reference: Normal)	Higher than reference range	0.89 (0.66–1.21)	0.4572	0.7394
	Missing	0.84 (0.20–3.56)	0.8076	
Aspartate aminotransferase (reference: Normal)	Higher than reference range	1.47 (1.10–1.97)	0.0101	0.0326
	Missing	1.58 (0.21–11.8)	0.6549	
International Normalised Ratio (reference: Normal)	Higher than reference range	0.90 (0.66–1.22)	0.4829	0.0059
	Missing	0.63 (0.48–0.84)	0.0014	

aBTC, advanced biliary tract cancer; ALBI, albumin-bilirubin; BTC, biliary tract cancer; CI, confidence interval; MASH, metabolic dysfunction-associated steatohepatitis; MASLD, metabolic dysfunction-associated steatotic liver disease.

Table S7. Sensitivity analysis: Cox proportional hazards model for real-world OS in patients with aBTCs treated with gemcitabine-based chemotherapies

		Multivariate model		
**Parameter**	**Categories**	**Hazard ratio (95% CI)**	***p*-value**	**Type 3 *p*-value**
Age at index (years)	Continuous	1.02 (1.00–1.03)	0.0309	0.0309
Gender (reference: Male)	Female	0.87 (0.66–1.16)	0.3534	0.3534
Race (reference: White)	African American	0.86 (0.56–1.32)	0.4788	0.6542
	Other	0.83 (0.47–1.47)	0.5285	
Site of primary tumour (reference: Intrahepatic cholangiocarcinoma)	Extrahepatic cholangiocarcinoma	0.74 (0.48–1.16)	0.1913	0.0410
	Gallbladder cancer	1.34 (0.86–2.11)	0.1999	
	Ampulla of Vater cancer /BTC not otherwise specified	0.49 (0.28–0.86)	0.0132	
	Multiple sites	0.90 (0.43–1.90)	0.7845	
**Comorbid conditions of interest and risk factors of BTC**				
Biliary obstructive conditions (reference: No)	Yes	0.94 (0.68–1.32)	0.7387	0.7387
Cardiovascular disease (reference: No)	Yes	0.88 (0.65–1.19)	0.4106	0.4106
Diabetes (reference: No)	Yes	1.12 (0.83–1.53)	0.4497	0.4497
Liver cirrhosis (reference: No)	Yes	0.89 (0.54–1.46)	0.6377	0.6377
Obesity (reference: No)	Yes	0.89 (0.67–1.20)	0.4525	0.4525
**Conditions associated with the aetiology of BTC**				
Alcoholic liver disease (reference: No)	Yes	0.98 (0.47–2.05)	0.9543	0.9543

Hepatitis B virus (reference: No)	Yes	2.07 (0.70–6.18)	0.1910	0.1910
Hepatitis C virus (reference: No)	Yes	2.28 (1.07–4.83)	0.0319	0.0319
MASLD/MASH (reference: No)	Yes	0.77 (0.51–1.14)	0.1874	0.1874
**Laboratory measures**				
ALBI grade (reference: Grade 1)	Grade 2	1.91 (1.34–2.72)	0.0003	0.0015
	Grade 3	1.84 (1.11–3.04)	0.0173	
Alanine aminotransferase (reference: Normal)	Higher than reference range	0.96 (0.68–1.34)	0.8036	0.8036
Aspartate aminotransferase (reference: Normal)	Higher than reference range	1.36 (0.98–1.88)	0.0684	0.0684
International Normalised Ratio (reference: Normal)	Higher than reference range	0.81 (0.59–1.12)	0.1986	0.1986

aBTC, advanced biliary tract cancer; ALBI, albumin-bilirubin; BTC, biliary tract cancer; CI, confidence interval; MASH, metabolic dysfunction-associated steatohepatitis; MASLD, metabolic dysfunction-associated steatotic liver disease.

Figure S1. Patient selection criteria.

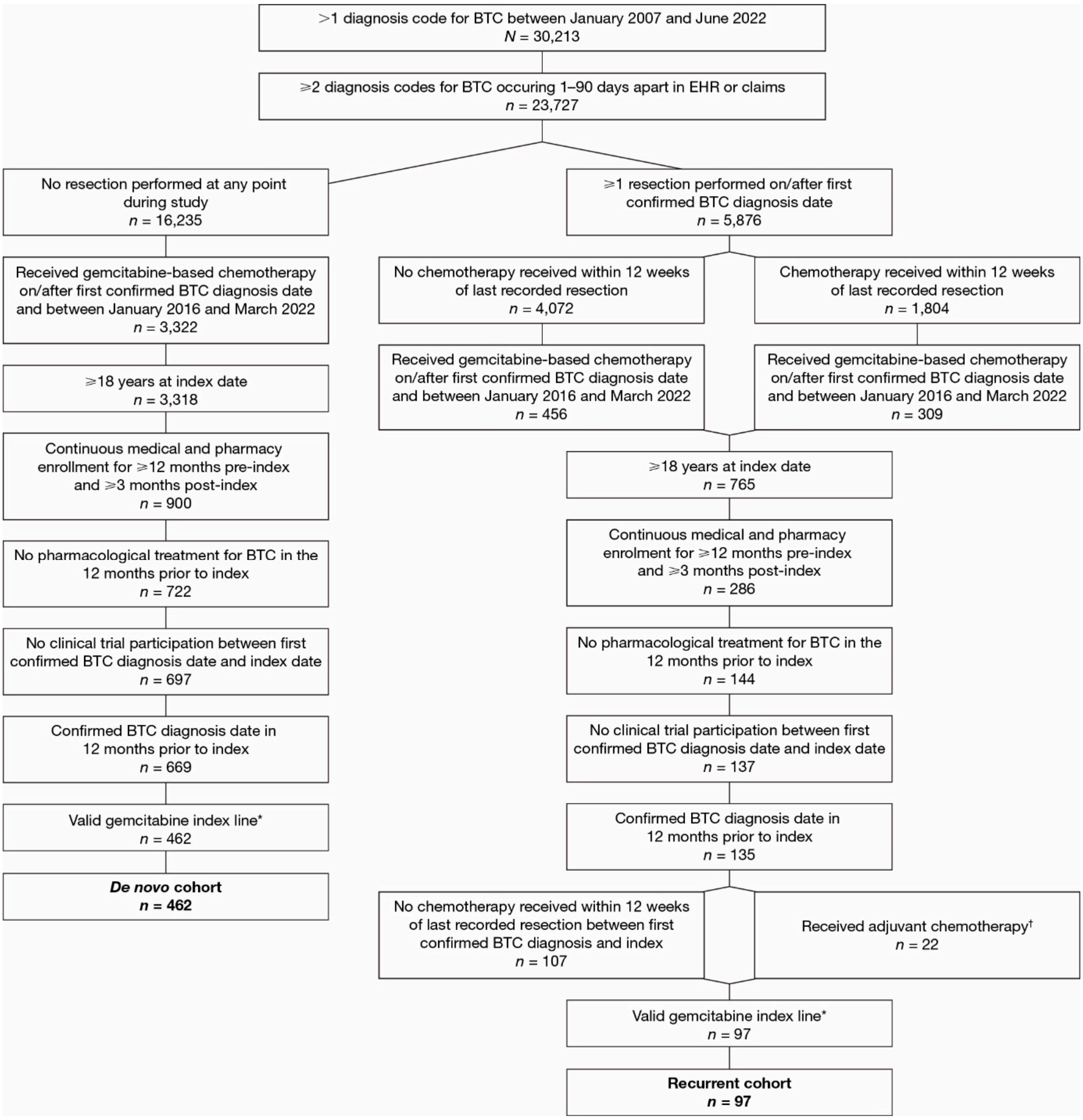

*Length of index line was ≥42 days (6 weeks); a line of therapy regimen was defined by all medications received within the first 30 days of the initiation of first-line gemcitabine for advanced BTC.

†Adjuvant chemotherapy must have lasted ≥12 weeks and ended ≥12 months prior to index.

BTC, biliary tract cancer; EHR, electronic health record.

Figure S2. Time from index to discontinuation or death by line of therapy in subgroups of patients with *de novo* or recurrent advanced biliary tract cancer. a Kaplan-Meier curves of time from index to discontinuation or death with first-line gemcitabine-based chemotherapy. **b** Kaplan-Meier curves of time from index to discontinuation or death with second-line therapy. **c** Kaplan-Meier curves of time from index to discontinuation or death with third-line therapy.

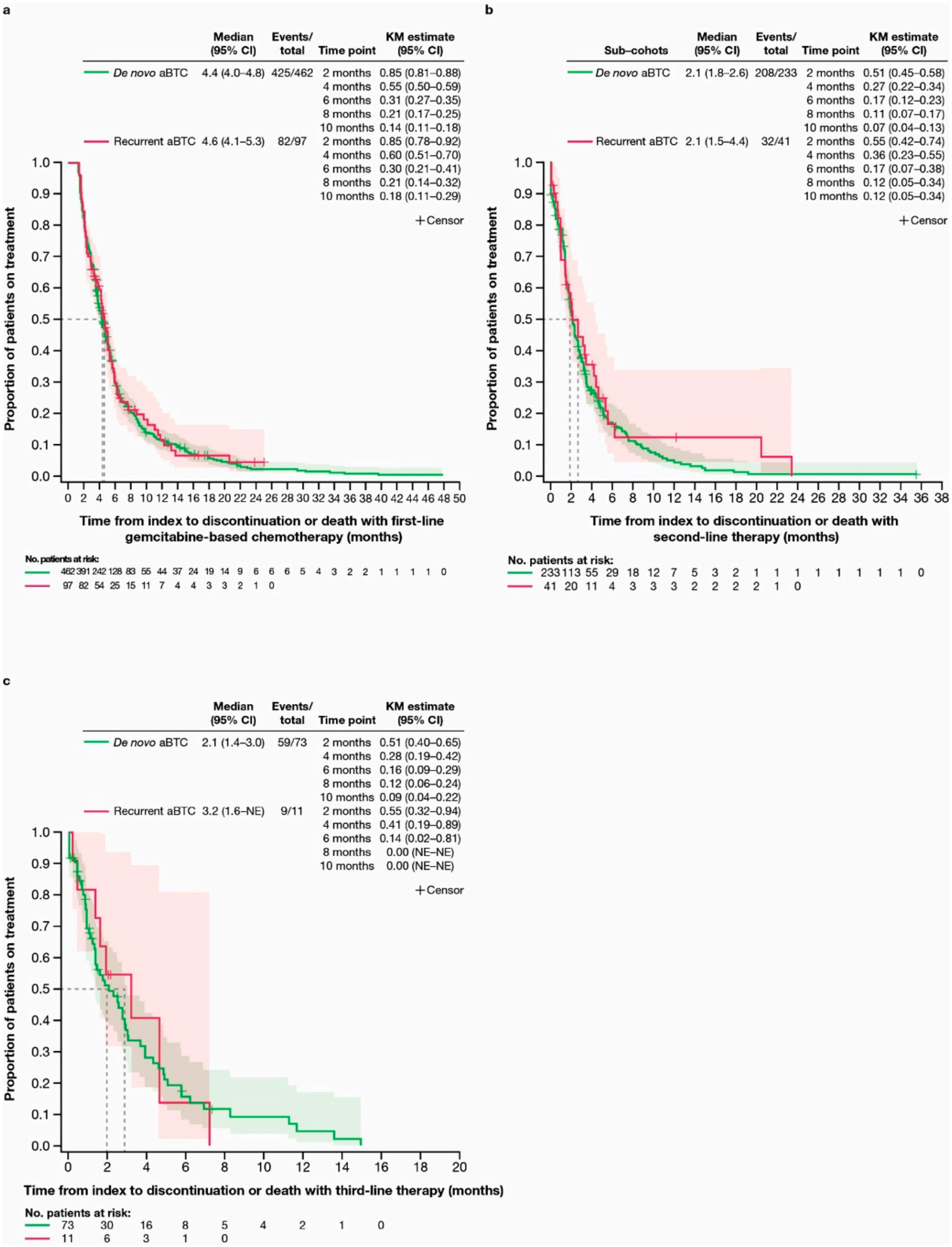

aBTC, advanced biliary tract cancer; CI, confidence interval; KM, Kaplan-Meier; NE, not evaluable.

Figure S3. Time from initiation of first-line gemcitabine-based chemotherapy or second-line therapy to subsequent line of therapy or death in subgroups of patients with *de novo* or recurrent advanced biliary tract cancer. a Kaplan-Meier curves of time from initiation of first-line gemcitabine-based chemotherapy to second-line therapy or death. **b** Kaplan-Meier curves of time from initiation of second-line therapy to third-line therapy or death.

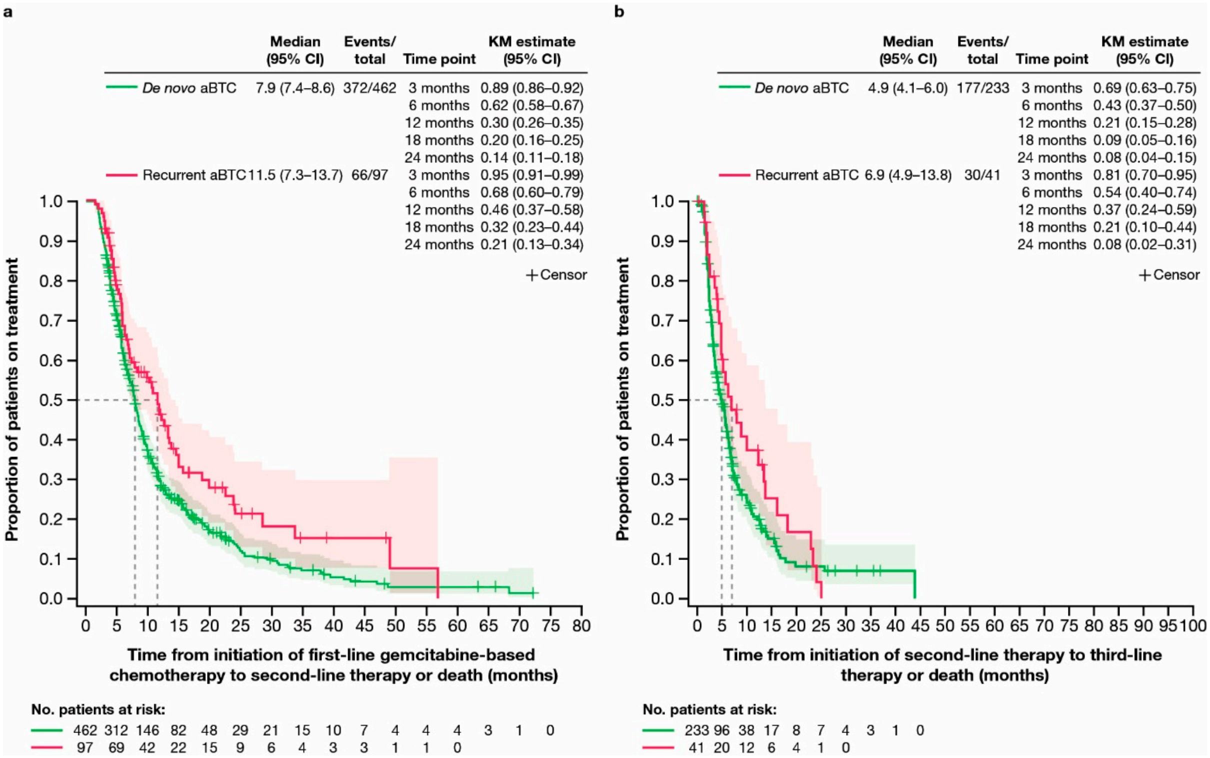

aBTC, advanced biliary tract cancer; CI, confidence interval; KM, Kaplan-Meier.
